# Comparison of robotic and laparoscopic rectal cancer surgery: a meta-analysis of randomized controlled trials

**DOI:** 10.1186/s12957-021-02128-2

**Published:** 2021-02-03

**Authors:** Bo Tang, Xiong Lei, Junhua Ai, Zhixiang Huang, Jun Shi, Taiyuan Li

**Affiliations:** 1 Nanchang University Medical College, Nanchang, Jiangxi Province China; 2 Department of General Surgery, The First Affiliated Hospital of Nanchang University, Nanchang, 330006 Jiangxi Province China

**Keywords:** Robotic surgery, Rectal cancer, Laparoscopic surgery, Postoperative complications

## Abstract

**Objective:**

Robotic and laparoscopic surgery for rectal cancer has been applied in the clinic for decades; nevertheless, which surgical approach has a lower rate of postoperative complications is still inconclusive. Therefore, the aim of this meta-analysis was to compare the postoperative complications within 30 days between robotic and laparoscopic rectal cancer surgery based on randomized controlled trials.

**Methods:**

Randomized controlled trials (until May 2020) that compared robotic and laparoscopic rectal cancer surgery were searched through PubMed, EMBASE, the Cochrane Library, China National Knowledge Infrastructure (CNKI), Wanfang Data Knowledge Service Platform, and China Biology Medicine disc (CBMdisc). Data regarding sample size, clinical and demographic characteristics, and postoperative complications within 30 days, including overall postoperative complications, severe postoperative complications (Clavien-Dindo score ≥ III), anastomotic leakage, surgical site infection, bleeding, ileus, urinary complications, respiratory complications, conversion to open surgery, unscheduled reoperation, perioperative mortality, and pathological outcomes, were extracted. The results were analyzed using RevMan v5.3.

**Results:**

Seven randomized controlled trials that included 507 robotic and 516 laparoscopic rectal cancer surgery cases were included. Meta-analysis showed that the overall postoperative complications within 30 days [*Z* = 1.1, OR = 1.18, 95% CI (0.88–1.57), *P* = 0.27], severe postoperative complications [*Z* = 0.22, OR = 1.12, 95% CI (0.41–3.07), *P* = 0.83], anastomotic leakage [Z = 0.96, OR = 1.27, 95% CI (0.78–2.08), *P* = 0.34], surgical site infection [*Z* = 0.18, OR = 1.05, 95% CI (0.61–1.79), *P* = 0.86], bleeding [*Z* = 0.19, OR = 0.89, 95% CI (0.27–2.97), *P* = 0.85], ileus [*Z* = 1.47, OR = 0.66, 95% CI (0.38–1.15), *P* = 0.14], urinary complications [*Z* = 0.66, OR = 1.22, 95% CI (0.67–2.22), *P* = 0.51], respiratory complications [*Z* = 0.84, OR = 0.64, 95% CI (0.22–1.82), *P* = 0.40], conversion to open surgery [*Z* = 1.73, OR = 0.61, 95% CI (0.35–1.07), *P* = 0.08], unscheduled reoperation [*Z* = 0.14, OR = 0.91, 95% CI (0.26–3.20), *P* = 0.89], perioperative mortality [*Z* = 0.28, OR = 0.79, 95% CI (0.15–4.12), *P* = 0.78], and pathological outcomes were similar between robotic and laparoscopic rectal surgery.

**Conclusion:**

Robotic surgery for rectal cancer was comparable to laparoscopic surgery with respect to postoperative complications within 30 days.

## Background

Laparoscopic rectal resection has been widely used for the treatment of rectal cancer because it results in a shorter length of hospital stay, less postoperative pain, and faster recovery of bowel function than open surgery [1–3]; however, laparoscopic technology is associated with some innate limitations, such as a two-dimensional view and limited dexterity, which may affect the surgery outcomes [4, 5].

Since robotic surgery was first used in rectal disease in 2001 [6], robotic surgery has gained great popularity worldwide. This technique has several advantages over laparoscopic surgery, including an immersive three-dimensional view of the surgical field, better dexterity capability, and a stable camera platform [7]. Surgeons hope that such innovative technology can alleviate some of the maneuverability and visibility challenges that surgeons encounter in narrow pelvic cavities.

A number of comparative studies have reported the results between robotic and laparoscopic surgery for rectal cancer, but it is still unclear which surgical approach has a lower rate of postoperative complications [8–10]. Therefore, we conducted this meta-analysis to evaluate the postoperative complications within 30 days between robotic and laparoscopic rectal cancer surgery based only on randomized controlled trials.

## Methods

### Search strategy

We conducted this meta-analysis in accordance with the Preferred Reporting Items for Systematic Reviews and Meta-analysis: the PRISMA statement [11].

The search strategy was according to the PICOT framework. P (population): adult population with primary rectal cancer; I (intervention): robotic rectal resection; C (comparison): laparoscopic rectal resection; O (outcomes): postoperative complication; and T (type of study design): randomized controlled trial. The following databases were searched: PubMed, EMBASE, the Cochrane Library, China National Knowledge Infrastructure (CNKI), Wanfang Data Knowledge Service Platform, and China Biology Medicine disc (CBMdisc). A systematic literature search was performed using the combination of medical subject headings (MeSH) and free-text words. The search terms were as follows: rectal neoplasm OR rectal cancer OR rectal carcinoma OR rectal tumor AND robotics OR robotic surgical procedures AND laparoscopy OR laparoscopic surgery AND randomized controlled trial OR prospective.

The last search was performed in April 2020, the search strategy was limited to papers written in English or Chinese, and the reference lists of the eligible studies were tracked manually for other potentially relevant studies.

### Eligibility criteria and study selection

Two independent authors (TB, HZX) screened the articles retrieved from the initial literature, duplicate studies were removed, and irrelevant studies were discarded. Two authors further reviewed the eligibility studies independently in abstract form or in full text by assessing if the eligibility criteria were met. Disagreements regarding study selection between the two authors were resolved by discussion and consensus or by consulting a third independent author (LX). Eligibility criteria were predetermined as follows: (1) randomized controlled trials, (2) comparison between robotic and laparoscopic surgery for resection of rectal cancer, and (3) clearly defined postoperative complications.

### Data extraction

The following data from the enrolled studies were extracted independently by two authors (TB, HZX). Discrepancies in data extraction between the two authors were resolved by discussion with the third author:

1. Characteristics of included studies

The publication year, country of the study, study design, operative methods, sample size, age, sex, body mass index (BMI), American Society of Anesthesiologists (ASA) grading, level of tumor from the anal verge, neoadjuvant therapies, robotic surgical technique, sphincter-saving procedures, diverting ileostomy and follow-up duration.

(2) Primary outcomes

Postoperative complications within 30 days included overall postoperative complications, severe postoperative complications (Clavien-Dindo score [12] ≥ III), anastomotic leakage, surgical site infection, bleeding (including intra-abdominal bleeding, intraluminal bleeding, and extra-abdominal bleeding), ileus, urinary complications, and respiratory complications.

(3) Secondary outcomes

Conversion to open surgery, TME completeness, number of harvested lymph nodes, proximal margin, distal margin, unscheduled reoperation, and perioperative mortality

### Risk of bias assessment

The quality of the included RCTs was evaluated using the Cochrane Collaboration’s tool for assessing risk of bias [13] and the Jadad score (low quality < 2, high quality ≥ 3) [14]. Discrepancies regarding the quality assessment of the included studies between the two authors were resolved by discussion with the third author.

### Statistical analysis

Review Manager (RevMan version 5.3, Copenhagen, Nordic Cochrane Center, Cochrane Collaboration, 2014) was used to perform the meta-analysis. Dichotomous variables were analyzed using the odds ratio (OR) with a 95% CI, and continuous variables were analyzed using the mean difference (MD) with a 95% CI; if continuous variables were reported as the median with range, we calculated the means and standard deviations according to Hozo et al. [15]. Heterogeneity was evaluated by the *I*^2^ statistic. If *I*^2^ < 50%, data analysis was performed by using a fixed effects model; otherwise, a random-effects model was used. *P* < 0.05 was considered statistically significant. Publication bias among the included studies was evaluated by funnel plots. Sensitivity analysis was performed by excluding studies with low methodological quality. The overall postoperative complications within 30 days were analyzed by trial sequential analysis.

## Results

### Literature searching

**Fig. 1:**
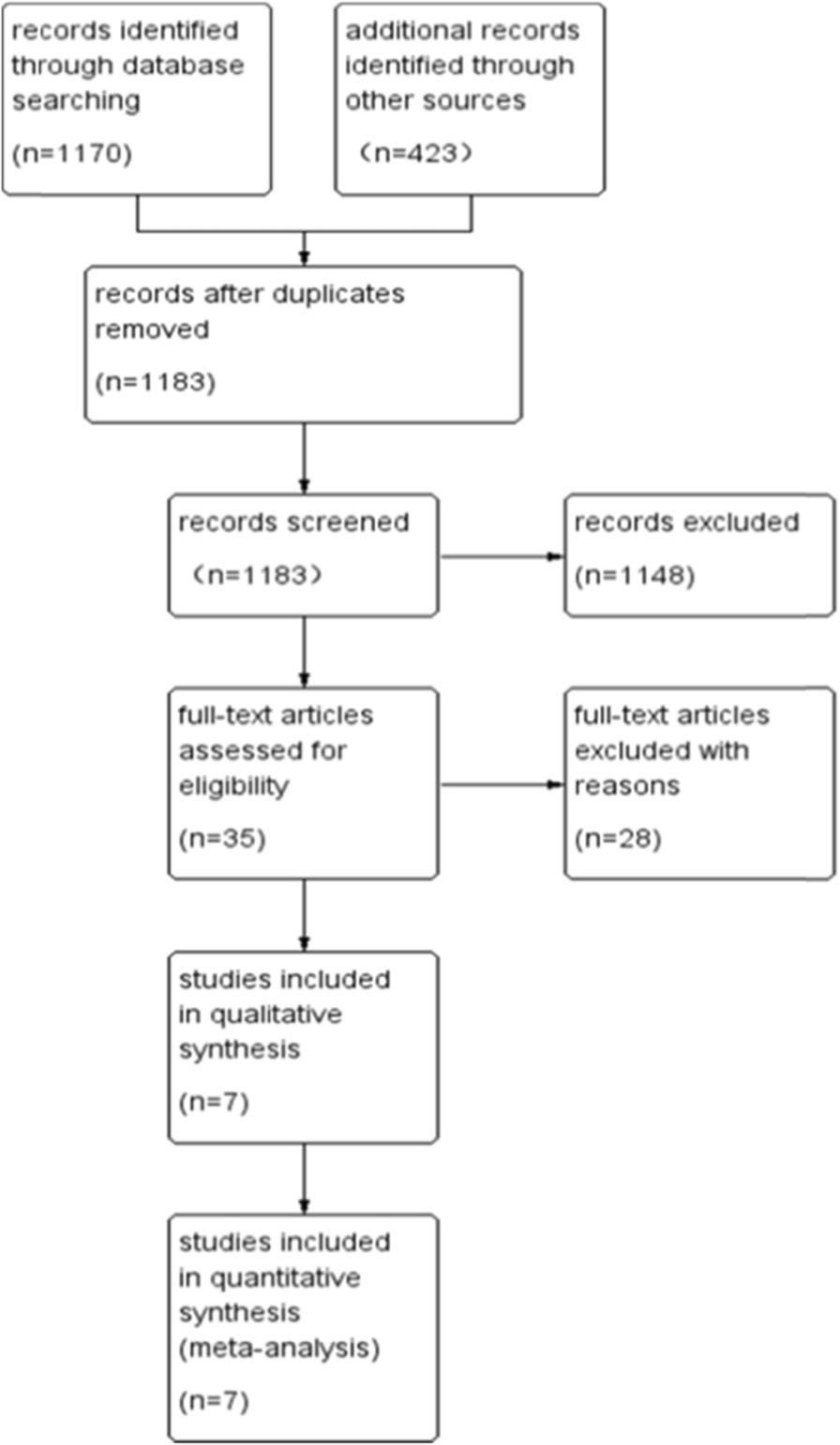
PRISMA flow chart of study selection

A total of 1593 studies were identified in the initial screening. After excluding duplicated studies, we screened 1183 studies and identified 35 eligible studies by scanning the title and abstract. Of these 35 studies, we identified seven articles that met the inclusion criteria for the final analysis after full-text evaluation [16–22]. The study selection progress is presented in Fig. 1.

### Characteristics of the included studies

The included studies involved 1023 patients (ranging from 36 to 471 per trial) from five countries (Korea, China, Egypt, UK, and Italy), with 507 patients in the robotic group and 516 patients in the laparoscopic group. The mean age ranged from 55.1 to 69 years, and the male to female ratio was 2.2:1. The mean BMI varied from 22 to 25.4 kg/m^2^. The patients of ASA I score account for 6 to 80%, ASA II accounts for 20 to 64%, ASA III accounts for 0 to 53%, and ASA IV accounts for 0 to 2.8%. The rate of neo-adjuvant therapies was 39.9% in the robotic group and 38.2% in the laparoscopic group. Six included studies reported the robotic surgery technique, of which three used a hybrid robotic surgery technique, two used a full-robotic surgery technique, and one used both a hybrid and full robotic surgery technique. The rate of sphincter-saving procedures was 85.2% in the robotic group and 84.5% in the laparoscopic group. The rate of diverting ileostomy was 49.9% in the robotic group and 52.7% in the laparoscopic group. Five studies described the follow-up duration. The characteristics of included studies are summarized in Table 1.

**Table 1:** Characteristics of the included studies

Study	Country	Study design	Group	Patients	Mean age	Sex (M/F)	Mean BMI	ASA (I/II/III/IV)	Tumor location from anal verge (< 5/> 5)	Neoadjuvant therapies	Robotic surgical technique	Sphincter-saving procedures	Diverting ileostomy	Follow-up duration (months)
Bailk et al. [21]	Korea	RCT	Rob	18	57.3 ± 6.3	14/4	22.8 ± 1.8	12/6/0/0	11.3 ± 2.5	-	Hybrid	18	0	-
			Lap	18	62.0 ± 9.0	14/4	24.0 ± 2.5	10/6/1/1	11.0 ± 2.5	-	-	18	0	-
Debakey et al. [17]	Egypt	RCT	Rob	21	53.4 (32–67)	11/10	-	18/3/0/0	2/19	12	Full-robotic	20	-	-
			Lap	24	50.3 (36–64)	13/11	-	18/6/0/0	3/21	11	-	21	-	-
Jayne et al. [19]	UK	RCT	Rob	237	64.4 ± 10.98	161/76	-	39/150/46/0	57/178	111	Hybrid+full-robotic	184	142	0–6
			Lap	234	65.5 ± 11.93	159/75	-	52/124/52/1	61/168	108	-	185	157	0–6
Kim et al. [16]	Korea	RCT	Rob	66	60.4 ± 9.7	51/15	24.1 ± 3.3	20/46/0/0	44/22	51	Hybrid	65	65	0–12
			Lap	73	59.7 ± 11.7	52/21	23.6 ± 3.0	30/43/0/0	55/18	58	-	71	70	0–12
Patriti et al. [20]	Italy	RCT	Rob	29	68 ± 10	11/18	24 ± 6.2	2/13/14/0	5.9 ± 4.2	7	Hybrid	24	-	29.2
			Lap	37	69 ± 10	12/25	25.4 ± 6.44	2/14/21/0	11 ± 4.5	2	-	34	-	18.7
Tang et al. [22]	China	RCT	Rob	65	55.1 ± 12.1	36/29	22 ± 2.5	35/30/0/0	6 ± 2.4	1	Full-robotic	52	15	9–31
			Lap	64	58.0 ± 9.7	36/28	22.1 ± 2.3	27/37/0/0	5.8 ± 2.6	0	-	44	13	9–31
Wang et al. [18]	China	RCT	Rob	71	60.3 (36–68)	71/0	22.9 (19–30)	-	46/25	13	Unknown	69	31	0–12
			Lap	66	58.7 (36–71)	66/0	22.4 (18–30)	-	40/26	11	-	63	32	0–12

### Risk of bias assessment

**Fig. 2:**
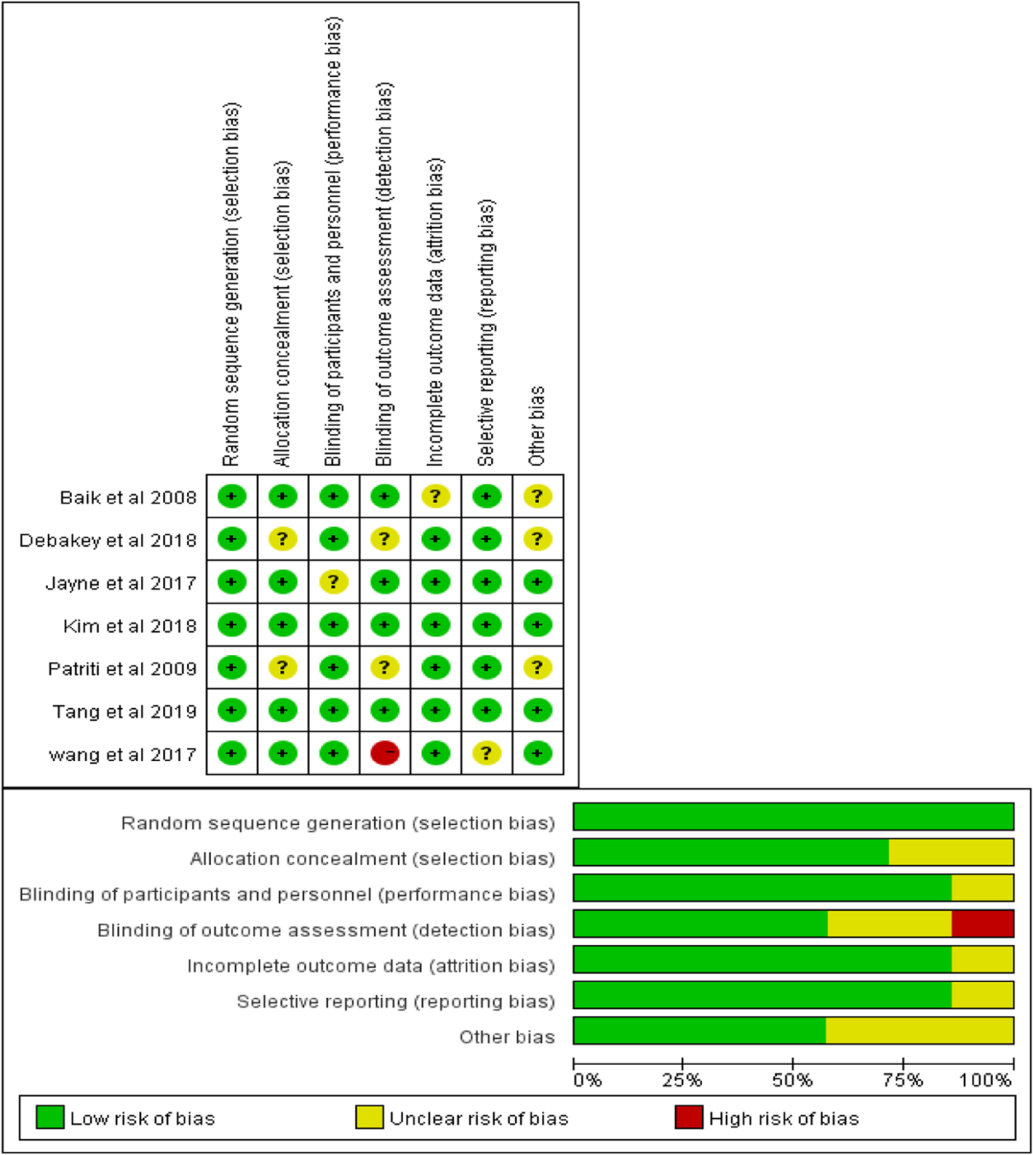
Risk of bias assessment of the included studies

Cochrane collaboration’s tool for assessing risk of bias indicated that all studies showed a lower risk of bias for random sequence generation. Regarding the blinding of participants and personnel, incomplete outcome data, and selective reporting, 86% of the RCTs were evaluated as low risk, and 14% were evaluated as unclear risk. The allocation concealment was evaluated as low risk in 71% of the RCTs, while 29% of the RCTs were evaluated as unclear risk. Regarding the blinding of outcome assessment, 57% of the RCTs were evaluated as low risk, 29% were evaluated as unclear risk, and one study was evaluated as high risk. Other biases were evaluated as low risk in four included studies and unclear risk in three included studies. Based on the risk of bias summary, two RCTs were considered to have a low risk of bias, four studies were considered to have an unclear risk of bias, and one study was considered to have a high risk of bias. The risk of bias assessment according to the Cochrane Collaboration’s tool is shown in Fig. 2.

The Jadad score showed that five included studies were considered high quality, whereas two included studies were considered low quality. Quality assessments of RCTs with Jadad scores are shown in Table 2.

**Table 2:** Quality assessments of RCTs with the Jadad score

Study	Randomization	Double blinding	Withdrawals and dropouts	Score summaries
Baik et al. [21]	2	0	1	3
Debakey et al. [17]	1	0	1	2
Jayne et al. [19]	2	0	1	3
Kim et al. [16]	2	0	1	3
Patriti et al. [20]	1	0	1	2
Tang et al. [22]	2	0	1	3
Wang et al. [18]	2	0	1	3

The full Jadad score was 5 points, and scores ≥ 3 were considered high quality

### Primary outcomes

#### Overall postoperative complications

**Fig. 3:**
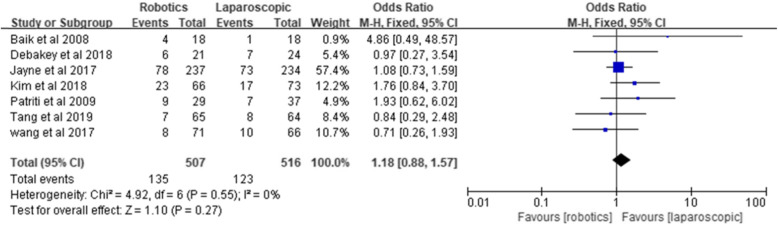
Overall postoperative complications

All the included studies compared overall postoperative complications. The overall postoperative complication rate was 26.6% in the robotic group and 23.8% in the laparoscopic group. The results of the meta-analysis suggested that there is no statistically significant difference in overall postoperative complications between robotic and laparoscopic rectal cancer surgery [*Z* = 1.1, OR = 1.18, 95% CI (0.88–1.57), *P* = 0.27], and no significant heterogeneity was found among the studies (*I*^2^ = 0%, *P* = 0.55) (Fig. 3).

#### Severe postoperative complications

**Fig. 4:**
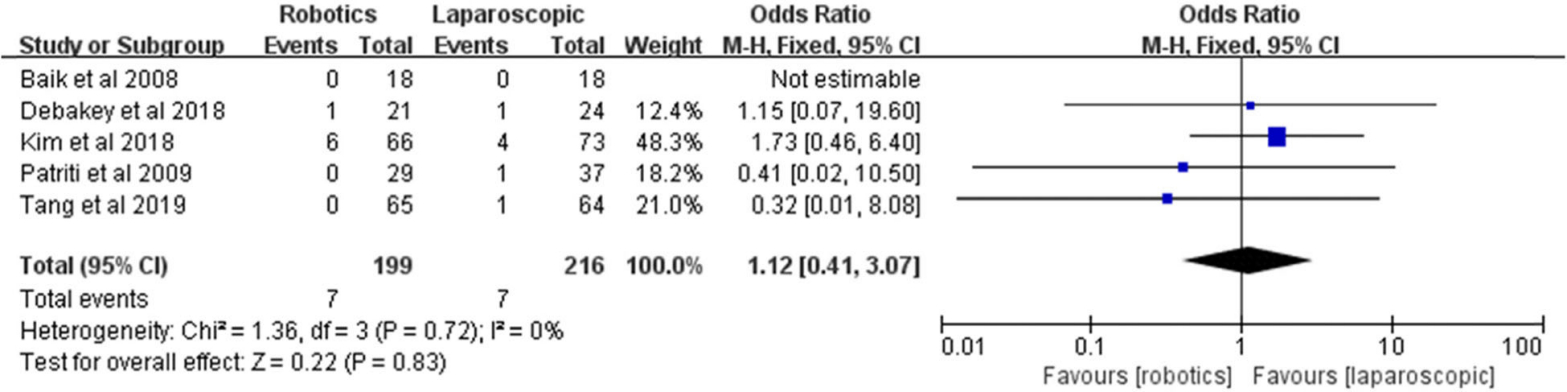
Severe postoperative complications

Five included studies compared the complications based on the Clavien-Dindo score. The rate of severe postoperative complications was 3.5% (7/199) in the robotic group and 3.2% (7/216) in the laparoscopic group. Pooled analysis showed no significant difference between the two groups [*Z* = 0.22, OR = 1.12, 95% CI (0.41–3.07), *P* = 0.83] and no heterogeneity among the studies (*I*^2^ = 0%, *P* = 0.72) (Fig. 4).

#### Anastomotic leakage

**Fig. 5:**
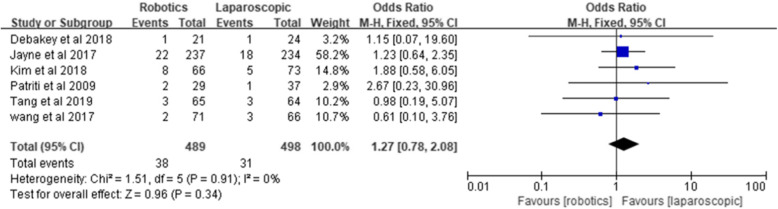
Anastomotic leakage

Six studies reported anastomotic leakage in 489 patients in the robotic group and 498 patients in the laparoscopic group. Pooling the six RCTs indicated no significant difference between the two groups [*Z* = 0.96, OR = 1.27, 95% CI (0.78–2.08), *P* = 0.34] and no heterogeneity (*I*^2^ = 0%, *P* = 0.91) (Fig. 5).

#### Surgical site infection

**Fig. 6:**
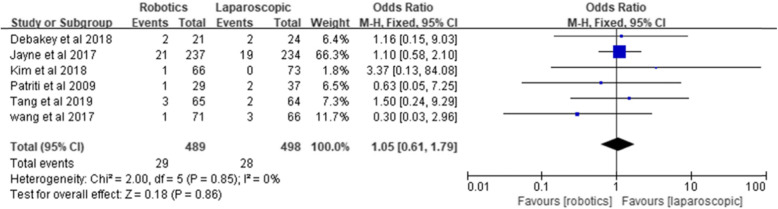
Surgical site infection

Six studies reported surgical site infection. The meta-analysis showed no significant difference between the two groups [*Z* = 0.18, OR = 1.05, 95% CI (0.61–1.79), *P* = 0.86] and no heterogeneity (*I*^2^ = 0%, *P* = 0.85) (Fig. 6).

#### Bleeding

**Fig. 7:**
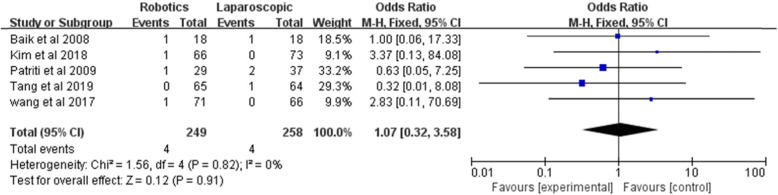
Bleeding

Bleeding was analyzed in five studies in a total of 249 robotic rectal surgeries and 258 laparoscopic rectal surgeries. No statistically significant difference was shown by the meta-analysis [*Z* = 0.12, OR = 1.07, 95% CI (0.32–3.58), *P* = 0.91], and the data did not show significant heterogeneity (*I*^2^ = 0%, *P* = 0.82) (Fig. 7).

#### Ileus

**Fig. 8:**
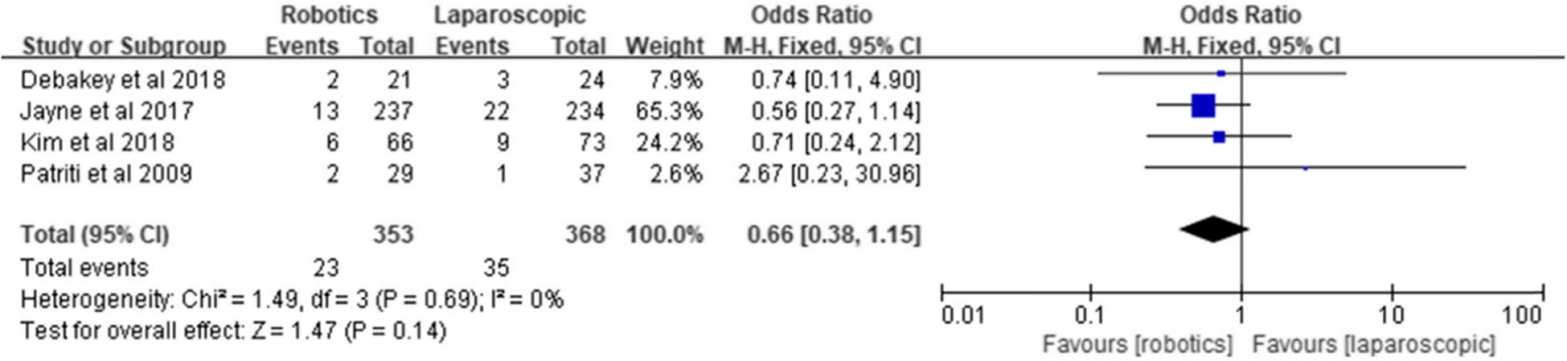
Ileus

Four studies reported ileus, involving 353 robotic surgeries and 368 laparoscopic surgeries. The results of the meta-analysis showed no significant difference between the two groups [*Z* = 1.47, OR = 0.66, 95% CI (0.38–1.15), *P* = 0.14] and no heterogeneity (*I*^2^ = 0%, *P* = 0.69) (Fig. 8).

#### Urinary complications

**Fig. 9:**
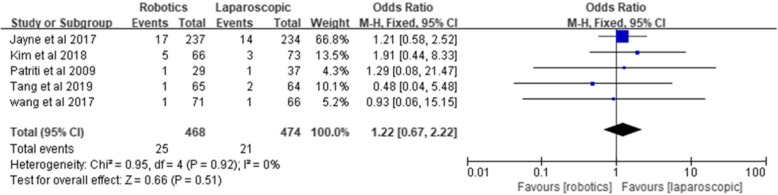
Urinary complications

Five studies reported urinary complications, involving 468 robotic surgeries and 474 laparoscopic surgeries. The incidence of urinary complications was 5.3% in the robotics group and 4.4% in the laparoscopic group. The pooled result showed no significant difference between the two groups [*Z* = 0.66, OR = 1.22, 95% CI (0.67–2.22), *P* = 0.51] and no heterogeneity (*I*^2^ = 0%, *P* = 0.92) (Fig. 9).

#### Respiratory complications

**Fig. 10:**
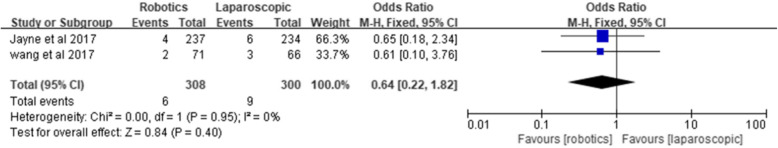
Respiratory complications

Only two studies reported respiratory complications. No statistically significant difference was found between the two groups [*Z* = 0.84, OR = 0.64, 95% CI (0.22–1.82), *P* = 0.40] and no significant heterogeneity among the studies (*I*^2^ = 0%, *P* = 0.95) (Fig. 10).

#### Conversion to open surgery

**Fig. 11:**
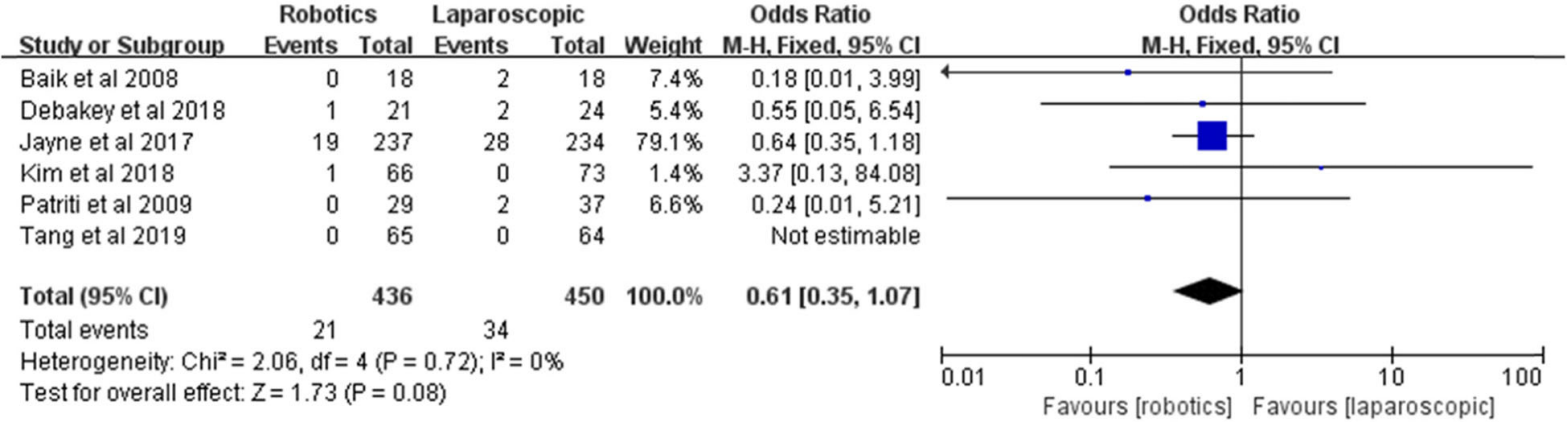
Conversion to open surgery

Six studies reported the conversion to open surgery. The rate of conversion to open surgery was lower in the robotic group than the laparoscopic group (4.8% vs. 7.6%), but the pooled results showed no statistically significant difference [*Z* = 1.73, OR = 0.61, 95% CI (0.35–1.07), *P* = 0.08] and no heterogeneity among the studies (*I*^2^ = 0%, *P* = 0.72) (Fig. 11).

### Secondary outcomes

#### TME completeness

**Fig. 12:**
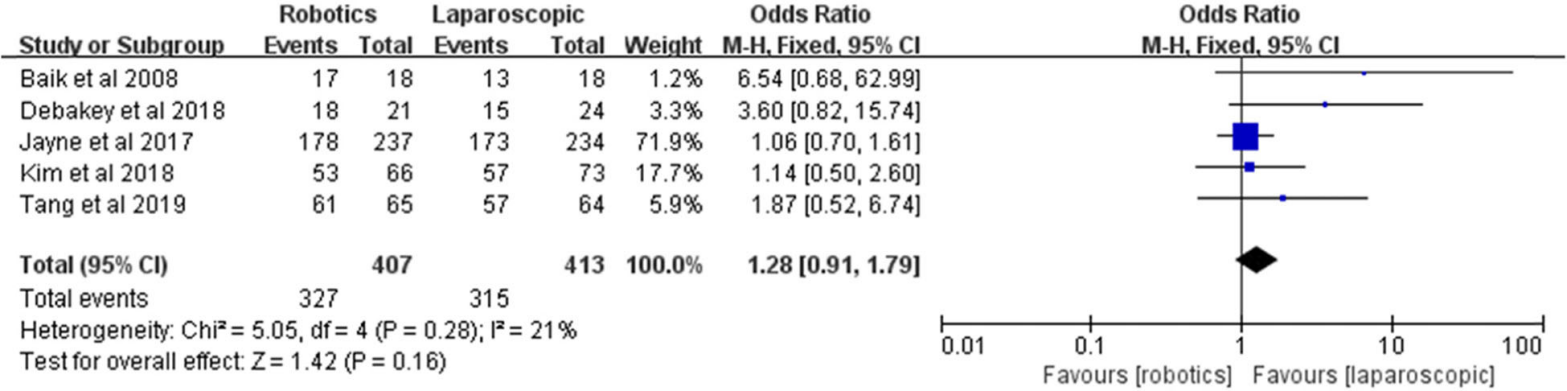
TME completeness

TME completeness was reported by five studies. The meta-analysis showed no difference between the two groups [*Z* = 1.42, OR = 1.28, 95% CI (0.91–1.79), *P* = 0.16] and no heterogeneity (*I*^2^ = 21%, *P* = 0.28) (Fig. 12).

#### Number of harvested lymph nodes

**Fig. 13:**
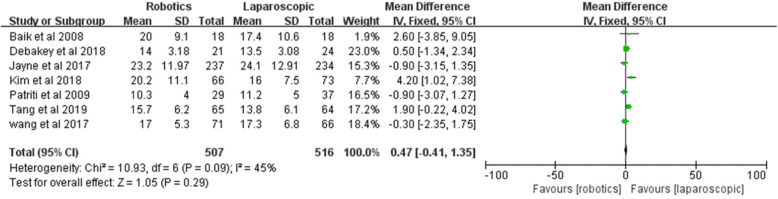
Number of harvested lymph nodes

All included studies reported the number of harvested lymph nodes. No significant difference was found between the two groups [*Z* = 1.05, MD = 0.47, 95% CI (− 0.41–1.35), *P* = 0.29]; 45% heterogeneity among the studies was observed (*I*^2^ = 45%, *P* = 0.09) (Fig. 13).

#### Proximal margin

**Fig. 14:**
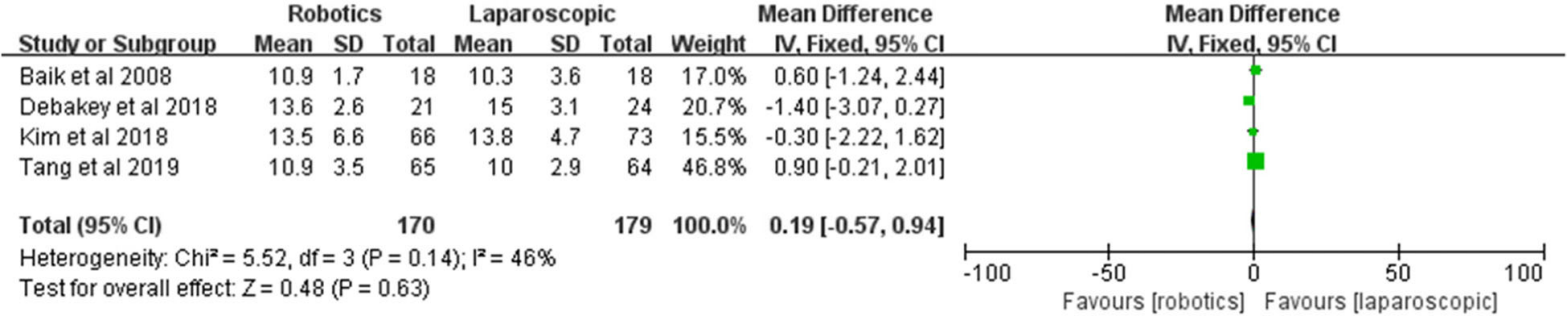
Proximal margin

The proximal margin was recorded in four studies. No significant difference was in the proximal margin between the two groups [*Z* = 0.48, MD = 0.19, 95% CI (− 0.57–0.94), *P* = 0.63]; 46% heterogeneity was found among the studies (*I*^2^ = 46%, *P* = 0.71) (Fig. 14).

#### Distal margin

**Fig. 15:**
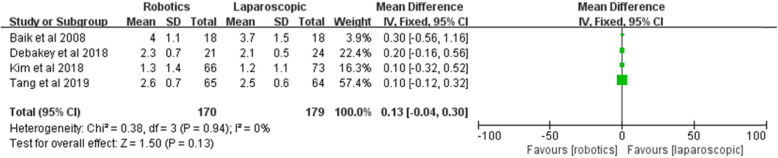
Distal margin

Four included studies described the distal margin. No significant difference in distal margins between the two groups [*Z* = 1.50, MD = 0.13, 95% CI (− 0.04–0.30), *P* = 0.13] and no heterogeneity among the studies (I^2^ = 0%, P = 0.0.94) were found (Fig. 15).

#### Unscheduled reoperation

**Fig. 16:**
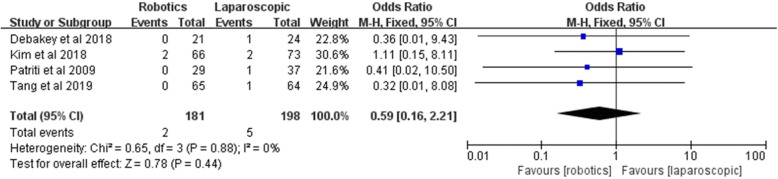
Unscheduled reoperation

Unscheduled reoperation was reported by four studies, involving 181 robotic surgeries and 198 laparoscopic surgeries. The meta-analysis showed no significant difference between the two groups [*Z* = 0.78, OR = 0.59, 95% CI (0.16–2.21), *P* = 0.44], and the data did not show significant heterogeneity (*I*^2^ = 0%, *P* = 0.88) (Fig. 16).

#### Perioperative mortality

**Fig. 17:**
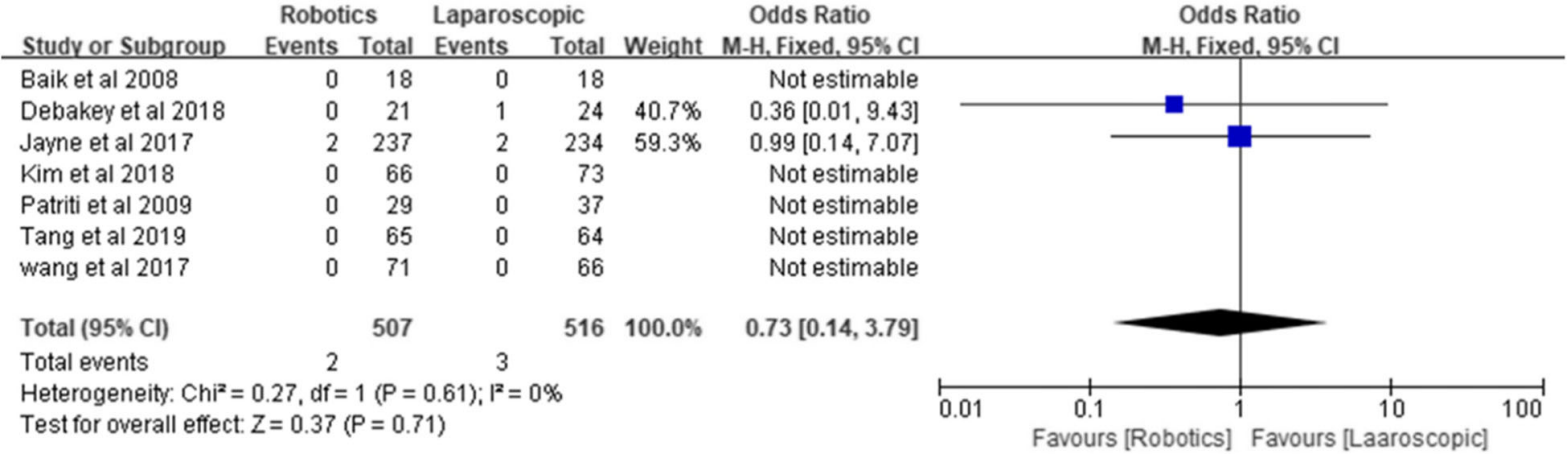
Perioperative mortality

Perioperative mortality was reported by all authors, including two perioperative deaths in the robotic group and three perioperative deaths in the laparoscopic group. Pooled perioperative mortality was similar: 0.39% for the robotic group and 0.58% for the laparoscopic group [*Z* = 0.37, OR = 0.73, 95% CI (0.14–3.79), *P* = 0.71], and no heterogeneity was observed (*I*^2^ = 0%, *P* = 0.61) (Fig. 17).

### Sensitivity analysis

**Fig. 18:**
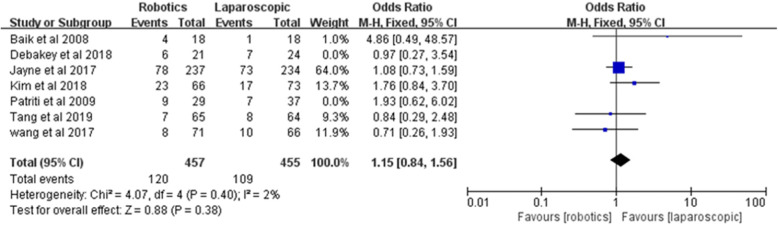
Sensitivity analysis of overall postoperative complications

Sensitivity analysis performed by excluding two studies [17, 20] with low quality (Jadad score ≤ 2) did not modify the pooled result of overall postoperative complications [*Z* = 0.88, OR = 1.15, 95% CI (0.84–1.56), *P* = 0.38; *I*^2^ = 2%, *P* = 0.40] (Fig. 18).

### Publication bias

**Fig. 19:**
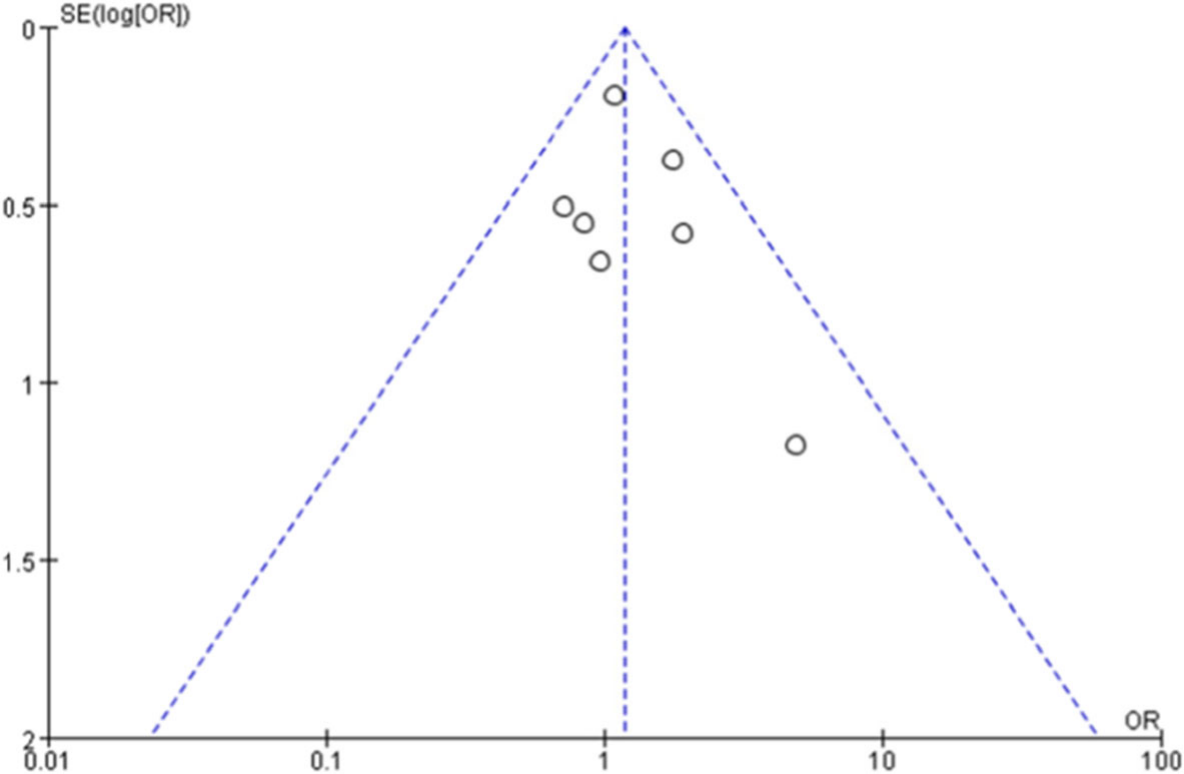
Funnel plot for overall postoperative complications

A funnel plot constructed for the overall postoperative complications showed that the possibility of publication bias was relatively small (Fig. 19).

### Trial sequential analysis (TSA)

**Fig. 20:**
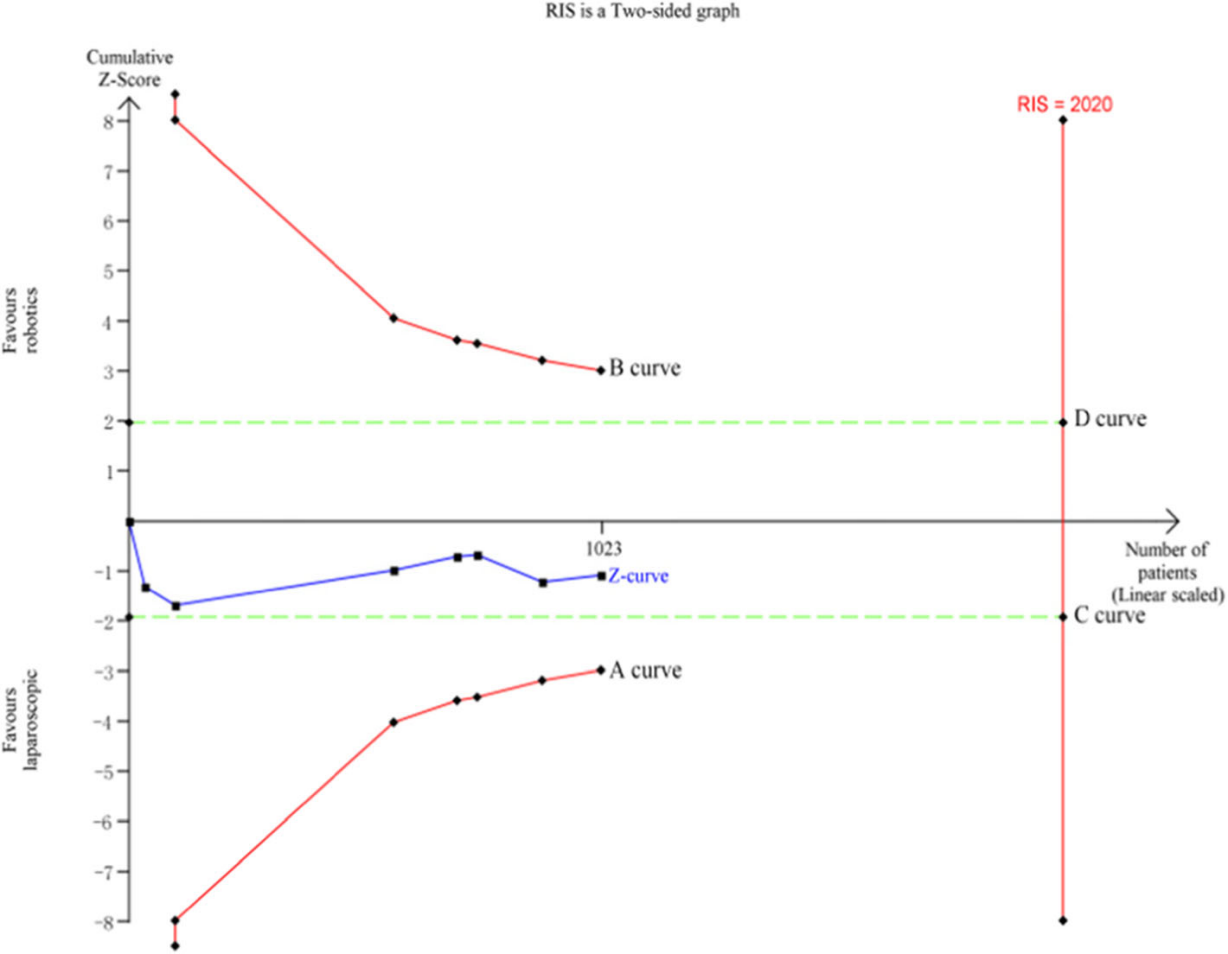
TSA for overall postoperative complications within 30 days

Using the TSA 0.9.5.10 Beta software, a total of 7 studies including 1023 cases were included for TSA according to the results of the meta-analysis of overall postoperative complications. The required information size (RIS) for the actual meta-analysis was 2020, and the estimation of RIS was based on the following statistical indicators: type I error rate (α = 0.05), type II error rate (*β* = 0.2), relative risk reduction (RRR = 20%), and incidence in the control arm (Pc = 26.6%). The TSA results showed that the cumulative *Z* value (*Z* curve) did not pass through the traditional text boundary (C and D curve) or crossed the TSA text boundary (A and B curve), and the cumulative information size did not reach the required information size (Fig. 20). Therefore, there may be no significant difference in overall postoperative complications between robotic and laparoscopic rectal cancer surgery, and more randomized controlled trials are needed to prove this finding.

## Discussion

With the development of laparoscopic technology, laparoscopic surgery has become a standard surgical procedure for rectal cancer. Compared to open surgery, laparoscopic surgery has the advantage of a shorter length of hospital stay, faster recovery, less postoperative pain, and earlier return to normal bowel function [23–25]; however, conventional laparoscopic rectal cancer surgery is technically demanding, especially for male and obese patients with a narrow pelvis and low rectal cancer. Laparoscopic rectal cancer surgery performed by a two-dimensional view and long straight instruments showed a higher conversion rate, which undoubtedly led to increased postoperative complications and worse oncological outcomes [26, 27]. Robotic surgery has the advantages of overcoming some innate limitations of laparoscopic surgery, including three-dimensional magnified vision, a stable camera platform, and better dexterity [28]. Although robotic surgery has been applied to the treatment of rectal cancer for decades, whether the advantages of the Da Vinci robot can result in better clinical benefits, such as a lower incidence of postoperative complications, remains debatable. Therefore, we designed this meta-analysis to answer this question.

In this meta-analysis, seven articles that met the inclusion criteria were included for the final analysis. Two RCTs were considered as having a low risk of bias, and the remaining studies were considered as having an unclear or high risk according to the Cochrane Collaboration’s tool because most of the studies did not report adequate information about the blinding of participants and personnel, incomplete outcome data, selective reporting, and allocation concealment regarding the blinding of outcome assessment. The Jadad score showed that five included studies were considered high quality, and two included studies were considered low quality. Because most of the included RCTs were open designs, double blinding was scored as 0 in all included studies. The double blinding score may influence the quality assessment of open RCTs; therefore, we included seven studies for further meta-analysis. The results of this meta-analysis showed that robotic rectal cancer surgery does not increase the incidence of postoperative complications within 30 days compared with laparoscopy.

Overall, postoperative complications within 30 days are an important index to measure the safety and feasibility of a surgical procedure. Therefore, we explored the advantages and disadvantages of robotic and laparoscopic surgery from the perspective of overall postoperative complications. Seven of the latest RCTs, including 507 patients undergoing robotic surgery and 516 patients undergoing laparoscopic surgery, were included, and the meta-analysis results showed no significant difference in overall postoperative complications between robotic rectal cancer surgery and laparoscopic surgery, which was consistent with previous RCTs and meta-analyses [10, 19, 29]; sensitivity analysis performed by excluding two studies with low quality and TSA also supported the results, and therefore, we concluded that robotic rectal cancer surgery is equally safe and feasible compared with laparoscopic surgery.

Anastomotic leakage is one of the most important complications after radical resection of rectal cancer. Acute diffuse peritonitis caused by anastomotic leakage is the most serious complication after rectal surgery and can lead to reoperation and even death [30]. In a previous study, the incidence of anastomotic leakage was 3.0 to 12.1% in robotic rectal surgery and 2.6 to 6.8% in laparoscopic surgery [16, 19] and was usually caused by a low anastomotic position, poor blood flow, high tension, and local infection [31, 32]. In this meta-analysis, the incidence of anastomotic leakage was 7.7% in the robotic group and 6.2% in the laparoscopic group. Most patients were treated conservatively, but two patients in the laparoscopic group underwent temporary ileostomy, and one patient died due to anastomotic leakage. The robotic group did not report the pooled results, and there was no significant difference in anastomotic leakage between the two groups, which is similar to the results of Prete et al. and Luo et al.’s meta-analysis [10, 29]. Hence, we concluded that robotic surgery for rectal cancer does not increase the occurrence of anastomotic leakage when compared with laparoscopic surgery.

Urinary complications are one of the parameters used to evaluate the protection of pelvic autonomic nerves during surgery. Although urinary complications are thought to be caused by multiple factors, iatrogenic damage during surgery is considered to be the main cause, which leads to a great negative impact on postoperative quality of life [33, 34]. Previous studies [18, 35] showed that robotic rectal surgery can significantly protect the pelvic autonomic nerve and reduce the incidence of postoperative urinary complications because of its 10-fold magnification of the surgical field; however, in our meta-analysis, a statistically significant difference in urinary complications between robotic and laparoscopic rectal surgery was not observed. Five included studies reported the results of urinary complications; however, two of the included studies did not have a clear definition of urinary complications, and there may be some clinical heterogeneity in the included studies. Therefore, further high-quality clinical research is needed to evaluate the advantages of robotic surgery in protecting the pelvic autonomic nerve.

The seven studies included in this meta-analysis incorporated the latest results of prospective randomized controlled trials, including the latest results of RCTs published by our center in April 2020 [22]. However, this current meta-analysis has certain limitations. First, detailed information concerning gastrointestinal complications, respiratory complications, and urinary complications was not mentioned clearly in the original studies, which may be one of the reasons for heterogeneity among the studies. Second, the sample size of some included studies was relatively small, and subgroup analysis was not performed in the original study, so the subgroup analysis in this meta-analysis was not conducted to explore the source of heterogeneity. Third, most of the included studies only reported short-term outcomes, and postoperative urinary and sexual function (questionnaires for International Prostate Symptom Score (IPSS) and International Index of Erectile Function (IIEF)), quality of life, and survival rate were not evaluated. Finally, the difference in comorbidities between the two groups, learning curve, surgeon experience, robotic surgical technique, the use of surgical instruments of different quality, and the measurement of results may produce some biases that are difficult to avoid and control.

## Conclusion

The present study suggested that robotic surgery for rectal cancer was comparable to laparoscopic surgery with respect to postoperative complications within 30 days; however, this meta-analysis is based on a very limited number of studies, two of which were published over a decade ago. Therefore, future high-quality multicenter RCTs are needed to confirm the advantage of robotic surgery for rectal cancer resection.

## Data Availability

Data sharing was not applicable to this article, as no datasets were generated or analyzed during the current study.

## Abbreviations

CNKI: China National Knowledge Infrastructure
CBMdisc: China Biology Medicine disc
MeSH: Medical subject headings
CI: Confidence interval
BMI: Body mass index
ASA: The American Society of Anesthesiologists
RCTs: Randomized control trials
OR: Odds ratio
MD: Mean difference
TSA: Trial sequential analysis
RIS: Required information size
